# Water–HCl Sequential Leaching of Waste Barrier Material from Aluminum Electrolysis Cell

**DOI:** 10.3390/ma18081748

**Published:** 2025-04-11

**Authors:** Yujie Zhao, Saiya Li, Junfeng Cheng, Yuting Chen, Weiping Liu, Wei Sun, Shafiq Alam

**Affiliations:** 1 School of Minerals Processing and Bioengineering, Central South University, Changsha 410083, China; 2 Engineering Research Center of Ministry of Education for Carbon Emission Reduction in Metal Resource Exploitation and Utilization, Central South University, Changsha 410083, China; 3 Department of Chemical and Biological Engineering, College of Engineering, University of Saskatchewan, Saskatoon, SK S7N 5A9, Canada

**Keywords:** lithium, waste barrier material, hydrochloric acid, selective leaching

## Abstract

The Hall–Héroult aluminum production process generates lithium-rich waste barrier materials, which are challenging to process using conventional acid leaching due to the environmental risks posed by hydrofluoric acid (HF) emissions. This research introduces a two-stage water–HCl sequential leaching (WHSL) approach to recover lithium while reducing these environmental impacts. The method evaluates key factors, such as the liquid–solid ratio, temperature, duration, rotation speed, and HCl concentration, and compares its efficacy with traditional HCl leaching using XRD, FTIR, DBP, and SEM techniques. The findings indicate that initial water leaching dissolves NaF salts, creating surface grooves and cracks. Subsequent HCl leaching selectively extracts lithium from aluminum and silicon, forming silica gel while preserving the nepheline phase due to its structural integrity. The process produces a porous residue with smaller particles, reduced surface potential, and promotes colloidal aggregation. This two-step process achieves efficient lithium recovery while reducing acid consumption and minimizing hydrogen fluoride (HF) emissions.

## 1. Introduction

Aluminum alloys, crucial for economic development [1], are primarily produced via the Hall–Heroult process in China [2]. Electrolytic cell overhaul slag, generated after a typical lifespan of 2500–3000 days, poses increasing environmental concerns. The composition of overhaul slag consists of various substances, including cathode carbon blocks, refractory materials, insulation materials, and waste barrier material. Waste barrier materials, which make up approximately 35% of the overhaul slag, contain relatively high lithium concentrations (1~3% Li). The effective separation of overhaul slag is essential for enhancing product quality and optimizing resource utilization. To achieve this, waste barrier material and cathode carbon block were concentrated from overhaul slags using a method that includes crushing, screening, and photoelectric sorting [3]. Despite significant research on lithium extraction from electrolytes [4], overhaul slag [5], and cathode carbon blocks [6], there is limited focus on the extraction and utilization of lithium from waste barrier material, highlighting a critical gap in research.

The current lithium extraction processes from aluminum electrolytic cells primarily focus on lithium-containing electrolytes or treat overhaul slag directly. The main hydrometallurgical methods for extracting lithium from the electrolytes or overhaul slags include acid leaching [7], alkaline leaching [8], salt-assisted leaching [9,10], and roasting leaching [4,11,12]. Acid leaching is a widely used industrial method due to its simplicity and high lithium extraction efficiency.

Hydrochloric acid (HCl) has emerged as a popular choice for leaching industrial waste due to its cost-effectiveness and availability [13], making it a viable option for extracting lithium from waste barrier material. However, this approach presents several challenges, including low selectivity and the generation of harmful hydrogen fluoride gas, which can contribute to environmental pollution [14]. To address these issues, as well as the inefficient use of fluorine resources commonly associated with traditional acid-leaching methods, a water–HCl sequential leaching (WHSL) approach has been proposed. In this method, soluble fluoride salts are dissolved in water, thereby preventing the formation of HF gas and significantly reducing acid consumption during the processing of alkaline waste barrier materials. This strategy effectively mitigates the environmental impact while improving the efficiency of the leaching process.

This study focuses on the WHSL of waste barrier material, aiming to identify optimal parameters for both water leaching and hydrochloric acid leaching. The key parameters under investigation include the acid concentration, liquid-to-solid ratio, reaction temperature, rotation speed, and reaction time, all of which influence the leaching behavior of lithium (Li), aluminum (Al), silicon (Si), and fluorine (F). The changes in phase composition, particle size, and surface morphology of the waste barrier material before and after the leaching process are also examined. The findings are intended to provide both technical and theoretical support for the comprehensive utilization of waste barrier material.

## 2. Materials and Methods

### 2.1. Materials and Sample Preparation

**Figure 1:**
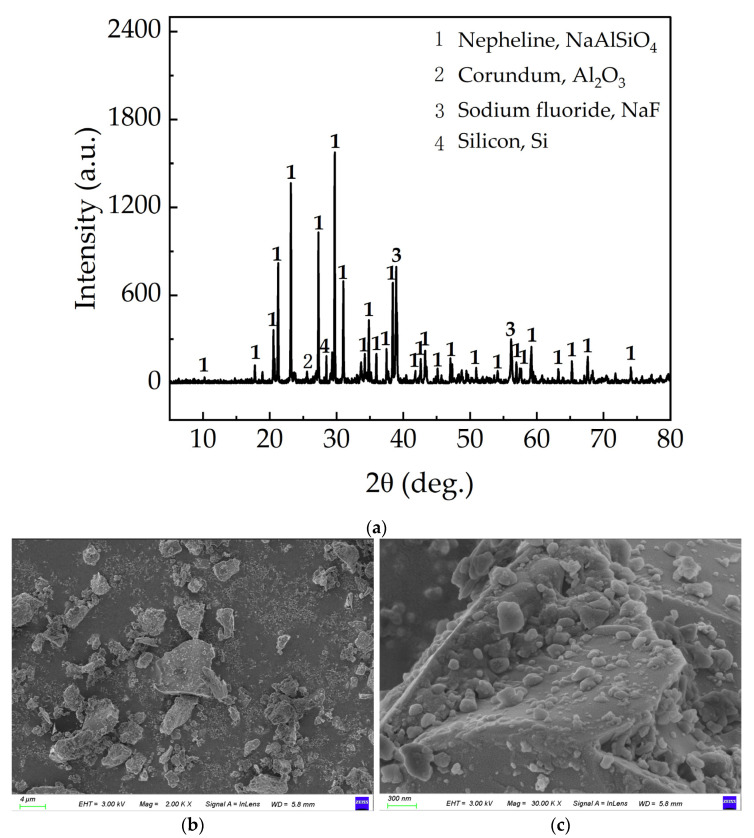
Phase and morphology analysis of waste barrier material: (**a**) XRD, (**b**) SEM-4 um scale, and (**c**) SEM-400 nm scale.

The waste barrier material used in this study was sourced from a sample provided by a solid waste utilization company in Qinghai Province. The materials had been pre-sorted using an intelligent sorting system. A comprehensive understanding of the physical, chemical, and mineralogical properties of waste barrier materials is crucial for their subsequent processing and utilization. Alumina refinery processing conditions, coupled with the diversity of bauxite sources and cell types, result in materials displaying a spectrum of colors, which range from gray-white and red-brown to black-gray, black, and white. The primary elemental composition of the material is shown in Table 1. The X-ray diffraction (XRD) analysis of the material is presented in Figure 1a, revealing that the main components are nepheline, corundum, sodium fluoride, and silicon. Microscopic analysis (Figure 1b,c) reveals that the waste barrier material primarily consists of irregularly shaped blocks, with its surface covered by numerous plate-like or spherical particles.

### 2.2. Experimental Methods

**Figure 2:**
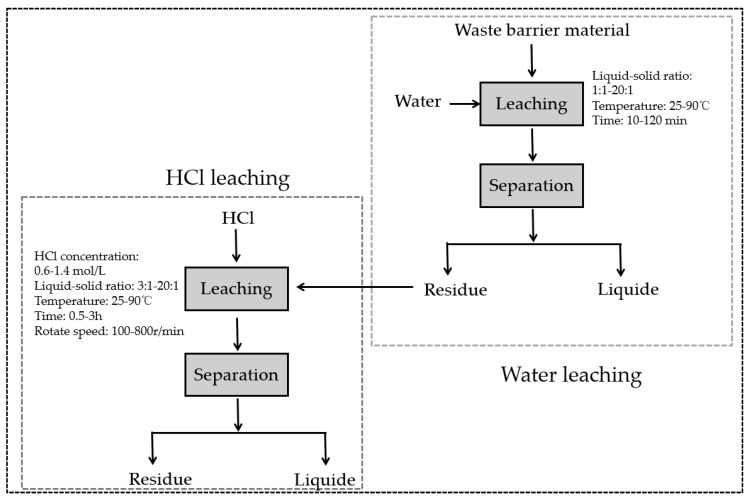
WHSL process of waste barrier material.

Leaching experiments were performed in 250 mL round-bottom flasks, placed in a thermostatic water bath (DF-101S, Shanghai Lichen Bangxi Instrument Technology Co., Shanghai, China) equipped with a magnetic stirrer. The rotation speed was maintained uniformly throughout the procedure to ensure effective mixing of solid particles with solution. A temperature probe monitored the reaction temperature, while the produced gas was neutralized with sodium hydroxide solution. Following the reaction, the mixture was filtered using a Buchner funnel to separate the solid and liquid phases. The solid phase from the initial filtration was washed with deionized water and re-filtered. The second filtrate was collected, and the solids were dried at 105 °C for 6 h before weighing. The full leaching process and parameters are depicted in Figure 2.

The element extraction rate was calculated as follows:

(1) $$\varepsilon =\frac{c_{1}V_{1}+c_{2}V_{2}}{W*m}*100\%$$

where ε is the element extraction rate (%);

c_1_ and c_2_ are the concentrations of the elements in the first and second filtrates (g/L);

V_1_ and V_2_ are the volumes of the first and second filtrates (L);

W is the element content in the material (%),

m is the mass of material used in each experiment (g).

### 2.3. Characterization

The chemical composition, phase composition, particle size distribution, and residue porous structure are critical factors in determining leaching performance and identifying potential bottlenecks for further improvement. In this regard, particle size analysis was conducted using laser diffraction (MASTER SIZER 2000, Malvern Panalytical, Malvern, UK). To quantify the concentrations of Li, Al, and Si in the raw material, leachate, wash solution, and residue, inductively coupled plasma optical emission spectroscopy (ICP-OES, iCAP 6300, Thermo Fisher Scientific, Waltham, MA, USA) was applied. X-ray diffraction (XRD, Bruker-axs D8 Advance, Berlin, Germany) was employed for solid material characterization, with a scan range of 10–80° at 40 kV and 20 mA. Surface morphology was examined using scanning electron microscopy (SEM, JEOL JSM-6490LV, Tokyo, Japan) coupled with energy-dispersive X-ray spectroscopy (EDS). Fluoride content was determined with a fluoride ion meter (PXSJ-270F, Shanghai Oushituo, Shanghai, China). The dibutyl phthalate (DBP) absorption capacity of the residue was assessed according to HGT 3072-2019 standards.

## 3. Results and Discussion

A thermodynamic analysis was conducted during the leaching process to understand the behavior of different components and address feasibility issues. The process began by using water to extract valuable elements from the waste barrier material, followed by hydrochloric acid for further extraction. The mechanism of the water-hydrochloric acid sequential leaching process was also systematically examined.

### 3.1. Thermodynamic Analysis of the Leaching Process

The waste barrier material predominantly consists of nepheline (NaAlSiO_4_), corundum (Al_2_O_3_), sodium fluoride (NaF), and silicon (Si). Li was undetectable in X-ray diffraction (XRD) analysis. The leaching experiments indicate that 10–20% of the Li is soluble in water, while 80–90% dissolves in hydrochloric acid. It is likely that Li exists in the form of soluble compounds, such as lithium fluoride (LiF) and/or lithium aluminosilicate (LiAlSiO_4_, an isomorph of nepheline) [15,16]. Elemental analysis identifies notable concentrations of F, Si, Li, and Al in the material. During acid leaching, F is liberated as hydrogen fluoride (HF), while Li and Al form soluble chlorides, including LiCl and AlCl_3_. Nepheline reacts with the acid to produce silicic acid (H_4_SiO_4_), which subsequently condenses into polysilicic acids (H_2_SiO_3_) and transforms into silica sols. These sols undergo gelation, ultimately forming silica gels (mSiO_2_·(m + 1)H_2_O), which contribute to a silicon-rich colloidal residue [17].

LiF(aq) + HCl(aq) = LiCl(aq) + HF(g)↑     ΔG^θ^ (90 °C) = −59.84 kJ/mol (2)

NaF(aq) + HCl(aq) = NaCl(aq) + HF(g)↑     ΔG^θ^ (90 °C) = −66.29 kJ/mol (3)

LiAlSiO_4_(s) + 4HCl(aq) = LiCl(aq) + AlCl_3_(aq) + SiO_2_(s) + 2H_2_O(l)     ΔG^θ^ (90 °C) = −310.37 kJ/mol (4)

NaAlSiO_4_(s) + 4HCl(aq) = NaCl(aq) + AlCl_3_(aq) + SiO_2_(s) + 2H_2_O(l)     ΔG^θ^ (90 °C) = −315.04 kJ /mol (5)

Al_2_O_3_(s) + 6HCl(aq) = 2AlCl_3_(aq) + 3H_2_O(l)     ΔG^θ^ (90 °C) = −418.74 kJ /mol (6)

Si (s) + 4HCl (aq) = SiCl_4_ (aq) + 2H_2_ (g)↑     ΔG^θ^ (90 °C) = −349.56 kJ/mol (7)

**Figure 3:**
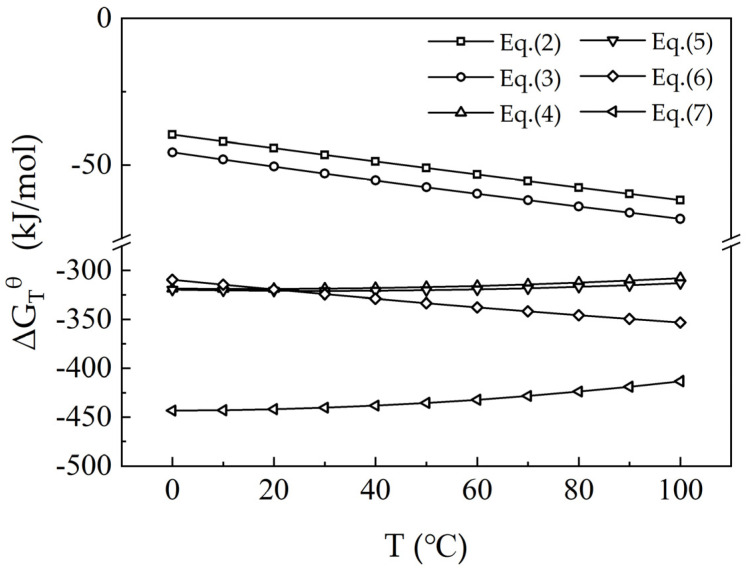
ΔG_T_^θ^ for reactions at various temperatures during HCl leaching.

The standard Gibbs free energy changes (ΔG^θ^) for the reactions were calculated in Figure 3. The results indicate that the ΔG^θ^ values for these reactions are negative at temperatures below 100 °C, indicating that these reactions are thermodynamically favorable. This suggests that hydrochloric acid (HCl) can effectively leach lithium-containing components, resulting in Li primarily existing in the leachate as chloride salt.

### 3.2. Influencing Factors in Water Leaching

**Figure 4:**
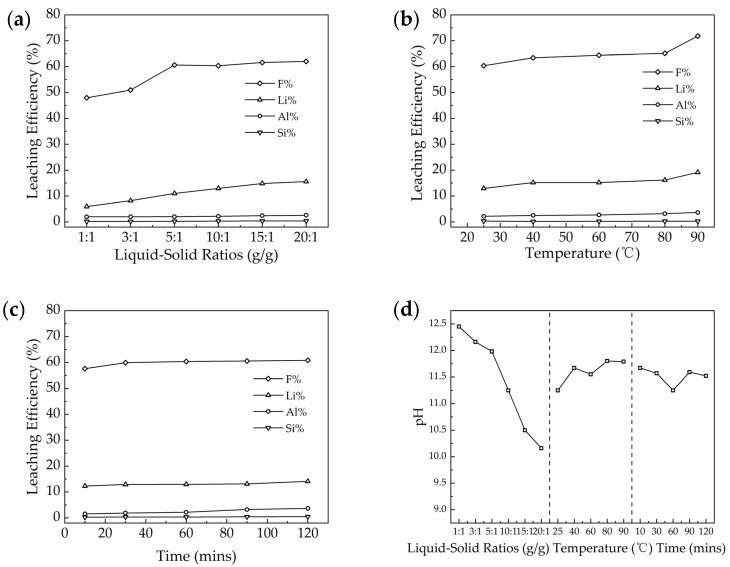
Influencing factors in water leaching of waste barrier material: (**a**) liquid–solid ratios, (**b**) temperatures, (**c**) leaching time, and (**d**) pH.

In response to the characteristics of the waste barrier material, which contains a significant amount of soluble NaF and exhibits alkaline properties (as shown in Figure 4d), the leaching experiments using water as the medium were conducted. This approach was chosen to prevent the neutralization of alkaline substances in the residue by acid during acid leaching, which could lead to the consumption of acid. This section analyzes and discusses the liquid-to-solid ratio, leaching temperature, and leaching time during the water-leaching process.

As illustrated in Figure 4a–c, the leaching rates for F and Li were approximately 50–70% and 5–20%, respectively, while the leaching rate for Al was around 2–4%, and for Si, it was about 0.2–0.4%. A higher liquid-to-solid ratio and elevated temperatures were beneficial for the leaching of F and Li, while the duration of leaching had minimal impact on the water leaching rates. As for the pH during leaching by water, the liquid–solid ratio had a significant impact on pH as expected; the higher liquid–solid ratio obtained a lower pH, while temperature and time had a limited impact on leaching pH.

The liquid–solid ratio, as depicted in Figure 4a, was examined. Increasing the amount of water dilutes the concentration of soluble substances in the aqueous solution, thereby enhancing the concentration gradient between the solid and liquid phases. This adjustment facilitates more efficient mass transfer, promoting leaching dynamics and the leaching efficiency. Additionally, a higher water volume facilitates better contact between solid particles and water, which is beneficial for increasing the leaching rate. It is evident that the liquid–solid ratio significantly affects the leaching rates of F and Li. As the liquid–solid ratio increases, the leaching rates for both substances improve. When the ratio reaches 5:1, the leaching rates for F and Li reach 60.6% and 11.0%, respectively. However, most Al- or Si-containing minerals are not soluble in water, which explains why the leaching rate for Al and Si remains relatively constant.

Increasing the leaching temperature enhances the solubility of soluble substances, such as NaF [18]. As demonstrated in Figure 4b, higher temperatures lead to an increased frequency of collisions between water molecules and soluble materials, thereby accelerating the leaching rate. The leaching rates of F and Li all improve with higher temperatures. When the temperature reaches 90 °C, the leaching rates for F and Li reach 71.8% and 19.2%, respectively. As illustrated in Figure 4c, the leaching time has a limited impact, and the majority of elements leached in 10 min; the F and Li leaching rates achieved 57.6% and 12.3%, respectively.

The waste barrier material exhibits alkaline properties, with leachate pH values ranging from 10 to 12.5 in aqueous solutions. This high alkalinity leads to elevated acid demand during traditional acid-based extraction processes, as neutralizing the materials’ basicity requires additional acid input. The waste barrier material contains water-soluble NaF, which raises the leachate pH through mild alkalization, further enhanced possibly by Na_2_CO_3_. During aluminum electrolysis, carbon cathodes degrade due to sodium oxidation and excess sodium, causing stress that transfers sodium carbonate into the surrounding waste barrier material, further elevating the pH during water leaching.

Water leaching primarily extracts lithium and fluoride. Based on the waste barrier’s phase composition (Figure 1), soluble NaF and LiF are primarily dissolved. Mild leaching conditions prevent gaseous fluoride evolution, ensuring all dissolved fluoride remains in the leachate. The liquid–solid ratio of 5:1, leaching temperature of 90 °C, and leaching time of 10 min were chosen in balance of cost and reaction efficiency.

### 3.3. Influencing Factors in HCl Acid Leaching

**Figure 5:**
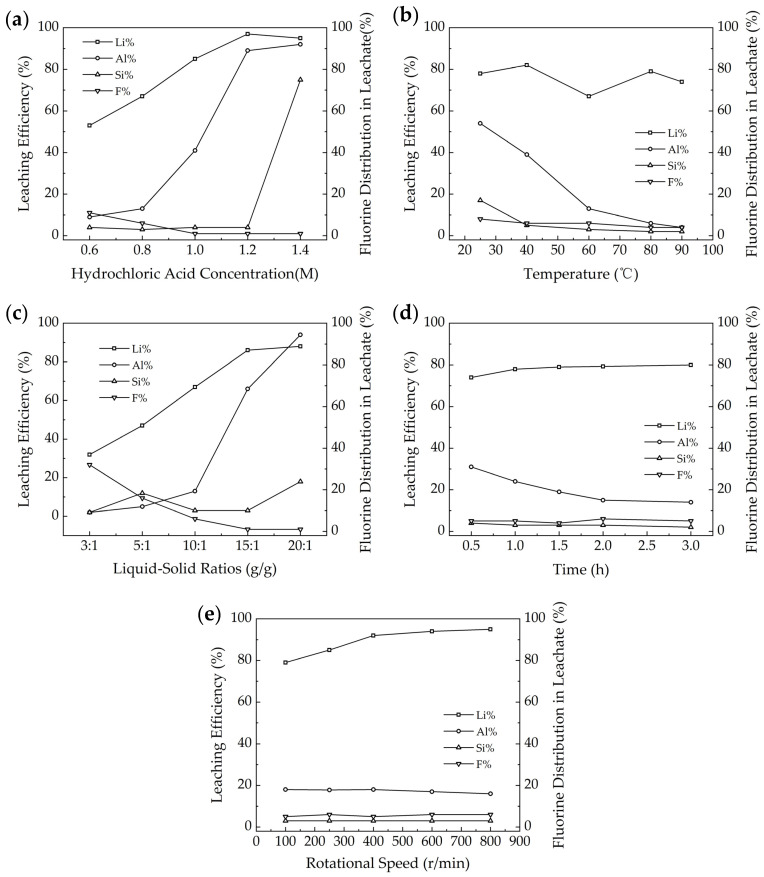
HCl leaching of waste barrier material: (**a**) HCl concentration, (**b**) temperature, (**c**) liquid–solid rations, (**d**) time, and (**e**) rotation speed.

The XRD analysis of waste barrier material (Figure 1) indicates that the main components are largely insoluble in water. This suggests that relying solely on water leaching to extract valuable elements from the waste barrier materials is not feasible. Furthermore, thermodynamic analysis (Equations (2)–(7)) reveals that the waste barrier material can react with hydrochloric acid, resulting in the formation of soluble salts. Therefore, key factors such as hydrochloric acid concentration, reaction temperature, liquid-to-solid ratio, leaching time, and rotation speed are examined to optimize the leaching efficiency, as shown in Figure 5. After the reaction, F in the waste barrier material was found in solid (residue), liquid (leachate), and gas phases. This section focuses solely on the distribution of F in the leachate. A comprehensive analysis of F distribution across all three phases is provided in Section 3.4.

As the concentration of HCl increases in Figure 5a, the leaching efficiency of Li and Al also increases, while the extraction rate of Si keeps constant below 1.4 mol/l, and fluoride distribution in leachate decreases. For optimal selective leaching of Li with minimal leaching of other elements, an HCl concentration of 0.6 mol/L is recommended. At this concentration, Li leaching efficiency reached 53%, while Al and Si leaching efficiencies were 9% and 4%, respectively.

The reaction temperature has a limited impact on Li leaching efficiency in Figure 5b, which varies between 67 and 82% as a function of reaction temperature. The Al and Si leaching efficiencies decreased from 54% and 17% at 25 °C to 4% and 2% at 90 °C, while F distribution in leachate varies in 4–8%.

A higher liquid–solid ratio leads to increased Li extraction rates, but it also results in higher extraction of Al in Figure 5c. Conversely, F in leachate decreases with higher liquid–solid ratios, while Si extraction is less than 10%. For optimal selective Li extraction with minimal co-leaching of other elements, a liquid–solid ratio of 10 is ideal. At this ratio, the extraction rates for Li, Al, and Si were recorded at 67%, 13%, and 6%, respectively, while the F distribution in leachate is 6%.

As shown in Figure 5d, the Li extraction rate varies in the range of 74–80% as a function of reaction time. The figure also demonstrates that the reaction time has little effect on the extraction of Si and F distribution in leachate, as their rates stabilize after 1 h (Li: 78%, Si: 5%, and F: 3%). However, the Al extraction rate decreases over time, reaching a minimum of 13% after 2 h. In summary, optimizing the reaction time is crucial for efficient Li extraction while minimizing the co-extraction of other elements like Si, F, and Al. In this regard, 2 h is selected as the optimum reaction time.

Overall, it seems the HCl concentration and liquid–solid ratio have a significant impact on Li leaching efficiency, while HCl concentration, temperature, and the liquid–solid ratio have a significant impact on Al leaching efficiency. The Si leaching efficiency and F distribution in leachate have limited variances as a function of the above parameters.

### 3.4. Water–HCl Sequential Leaching (WHSL)

Water leaching dissolved more than half of F, which avoids the reaction between F and HCl and its subsequent HF gas in the acid-leaching process. In this regard, WHSL was examined in this section. The waste barrier material was first leached by water, and then the residue from water leaching was subsequently leached by HCl. The optimum parameters based on Section 3.2 and Section 3.3 were used in sequential leaching, which aimed at selective leaching of Li and avoiding the HF gas. The WHSL was compared with water leaching and HCl leaching.

**Figure 6:**
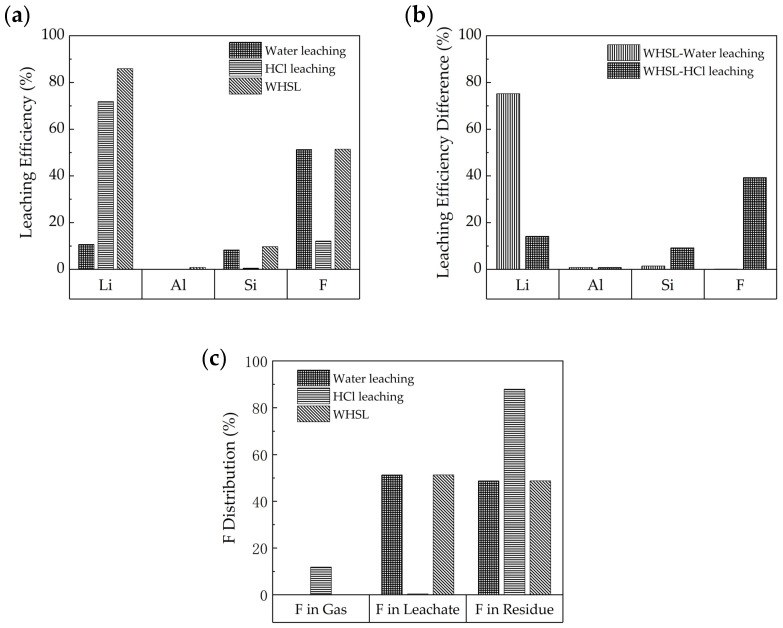
Comparison of water leaching, HCl leaching, and WHSL: (**a**) leaching efficiency, (**b**) leaching efficiency difference, and (**c**) F distribution.

The water leaching efficiencies of Li, Si, and F are 11%, 8%, and 51%, respectively, while the Li leaching efficiency in HCl leaching achieved 72%, as shown in Figure 6a. As for WHSL, the Li, Al, Si, and F leaching efficiencies are 86%, 1%, 10%, and 51%, respectively. As shown in Figure 6b, compared to water leaching, the Li leaching efficiency increased by 75%, while Al, Si, and F have limited change in WHSL. As compared to HCl leaching, the Li and F leaching efficiencies increased by 14% and 39%, respectively, in WHSL.

The F in the waste barrier material was distributed in solid, liquid, and gas phases in the HCl leaching process. The combination of F in the gas and liquid phases is equal to the F leaching efficiency. As shown in Figure 6c, the F mainly existed in the liquid phase with a distribution value of 51.3% in water leaching. As for HCl leaching, 11.8% of F was distributed in the gas phase, 0.3% of F existed in the liquid phase, and 87.9% of F in solid residue. The distribution of F in WHSL is the same as water leaching. It is obvious that WHSL avoided the F gas releasing and achieved selective extraction of Li from Al and Si when compared to the HCl leaching process.

### 3.5. Residue Characterization and Mechanism of WHSL

The waste barrier material underwent a WHSL process, beginning with water leaching, followed by HCl leaching. The residues from both stages were analyzed and compared with those from the HCl leaching process alone using various techniques, including X-ray diffraction (XRD), Fourier-transform infrared spectroscopy (FTIR), particle size analysis, dibutyl phthalate (DBP) absorption, and scanning electron microscopy (SEM).

#### 3.5.1. Surface Topography and Phase Characteristics

**Figure 7:**
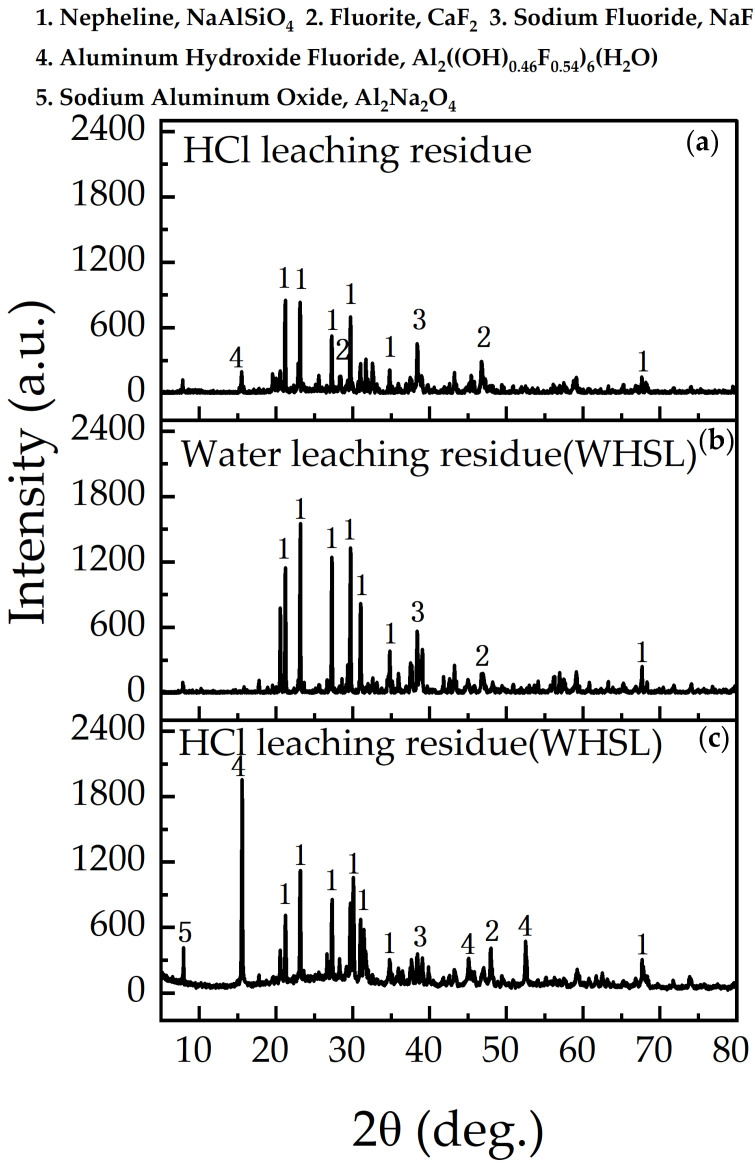
XRD of residue: (**a**) residue from HCl leaching, (**b**) residue from water leaching (WHSL), and (**c**) residue from HCl leaching (WHSL).

**Figure 8:**
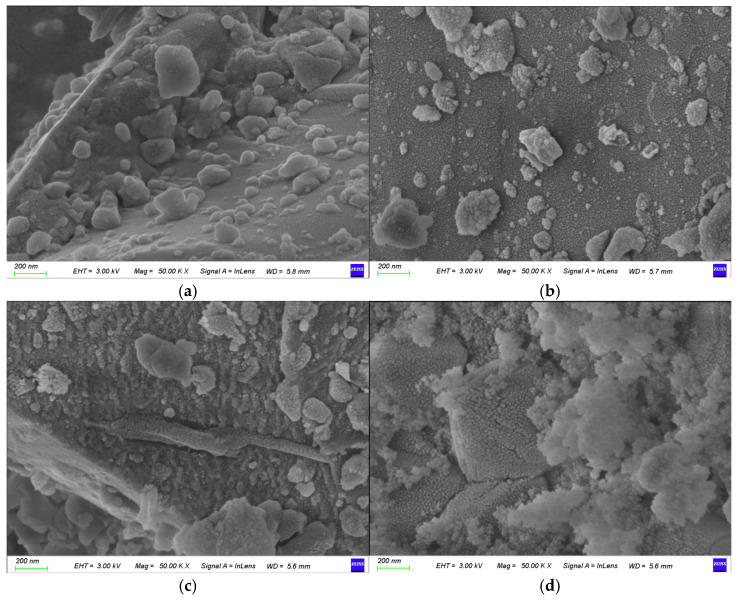
SEM results of leached residue: (**a**) waste barrier material, (**b**) HCl leaching, (**c**) water leaching (WHSL), and (**d**) HCl leaching (WHSL).

The residues from HCl leaching alone, as well as from the first (water leaching) and second (HCl leaching) stages of sequential leaching (HWSL), were analyzed using X-ray diffraction (XRD, Figure 7) and scanning electron microscopy (SEM, Figure 8). Compared to the waste barrier material (Figure 1a), the nepheline peak intensity in Figure 7a,c decreased as a result of its reaction with the acid, while the intensity in Figure 7b remained unaffected by water leaching. Compared to Figure 1a, the lower NaF peak density in Figure 7 suggests unreacted NaF may be trapped in solid particles.

After HCl leaching (Figure 7a,c), aluminum hydroxide fluoride and sodium aluminum oxide were detected, with stronger peak intensities in Figure 7c, particularly for aluminum hydroxide fluoride. Sodium aluminum oxide, usually found in the top layer of the waste barrier material [19], was not observed in Figure 1a, possibly due to its encapsulation in a dense phase [20]. However, following acid leaching, entrained materials were dissolved, thus revealing a stronger presence of sodium aluminum oxide in Figure 7a,c. Aluminum hydroxide fluoride, which forms in acid leachates with high aluminum and fluoride concentrations [21], was present in the acid-leaching residues (Figure 7a,c). CaF_2_ was also detected, which likely precipitated from the dissolved calcium and fluoride ions in the leaching solution. Aluminum, calcium, and silicon ions released during acid leaching react and precipitate in the residue, completing a “dissolve-precipitation cycle”.

SEM images of the waste barrier material and the residues subjected to various leaching treatments are shown in Figure 8. The waste barrier material (Figure 8a) consists mainly of block-like structures with smooth, spherical surfaces. After HCl leaching (Figure 8b), the residue exhibits a slightly rougher texture, likely due to the dissolution of nepheline, corundum, and sodium fluoride. The residue from the water leaching (WHSL, Figure 8c) also displays a roughened surface with distinct grooves, probably resulting from sodium fluoride dissolution. HCl leaching (WHSL) (Figure 8d) reveals a further roughened surface with white, flaky deposits, which are likely silica gel or aluminum fluoride hydroxide formed during acid treatment. XRD data (Figure 7) corroborate the formation of aluminum hydroxide fluoride, thereby supporting the observed morphological changes. Both HCl leaching and HCl leaching (WHSL) induce significant surface changes, including a loss of compactness and the formation of cracks.

#### 3.5.2. FTIR Analysis

**Figure 9:**
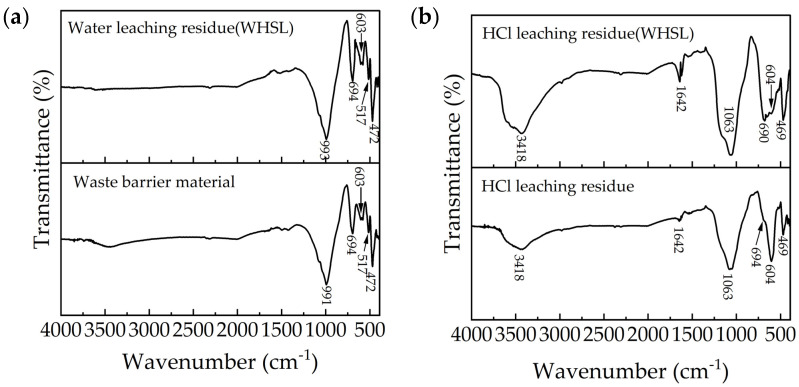
FTIR results from waste barrier material and leached residue in different conditions, (**a**) water leaching residue(WHSL) and waste barrier material, (**b**) HCl leaching residue(WHSL), HCl leaching residue.

FTIR spectroscopy was used to analyze the molecular composition and functional groups of materials based on their infrared absorption spectra, providing valuable insights into chemical structures and interactions. Figure 9 displays the Fourier-transform infrared spectroscopy (FTIR) spectra of the waste barrier material and the residues from HCl leaching and WHSL. The spectrum of the waste barrier material and the residue from water leaching (WHSL) (Figure 9a) shows distinct absorption peaks near 991, 694, 603, and 472 cm^−1^, attributed to nepheline [22]. The Si-O-Al band of short-range ordered aluminosilicates appears between 975 and 1018 cm^−1^ [23], with the peak near 472 cm^−1^ corresponding to the symmetric stretching and bending of the Si-O bond [24].

For the residue after HCl leaching and HCl leaching (WHSL) (Figure 9b), absorption peaks are observed at approximately 3418, 1642, 1063, 689, 604, and 469 cm^−1^. The bands in the 3500–3300 cm^−1^ range are indicative of hydroxyl groups (O-H stretching) and silanol groups (Si-OH), the latter mainly located on the surface of silica particles [25]. The band at 1643 cm^−1^ is associated with the bending mode of molecularly coordinated water adsorbed to the Si-O-Si structure [26]. The peak at 1063 cm^−1^ corresponds to amorphous silica [27], while the Si-O-Si band for amorphous silica typically appears between 1000 and 1100 cm^−1^ [28]. The peaks at 689, 604, and 469 cm^−1^ suggest the persistence of nepheline after leaching, in agreement with the XRD data (Figure 7).

The absorption band at 3418 cm^−1^ in the residue after HCl leaching (WHSL) (Figure 9b) is notably stronger than in the residue from sole HCl leaching, which aligns with the prominent peak of aluminum hydroxide fluoride observed in Figure 7c. Overall, FTIR indicates that amorphous silica gel and aluminum hydroxide fluoride were observed in the residue after HCl leaching and HCl leaching (WHSL).

#### 3.5.3. Particle Size and DBP Analysis

**Figure 10:**
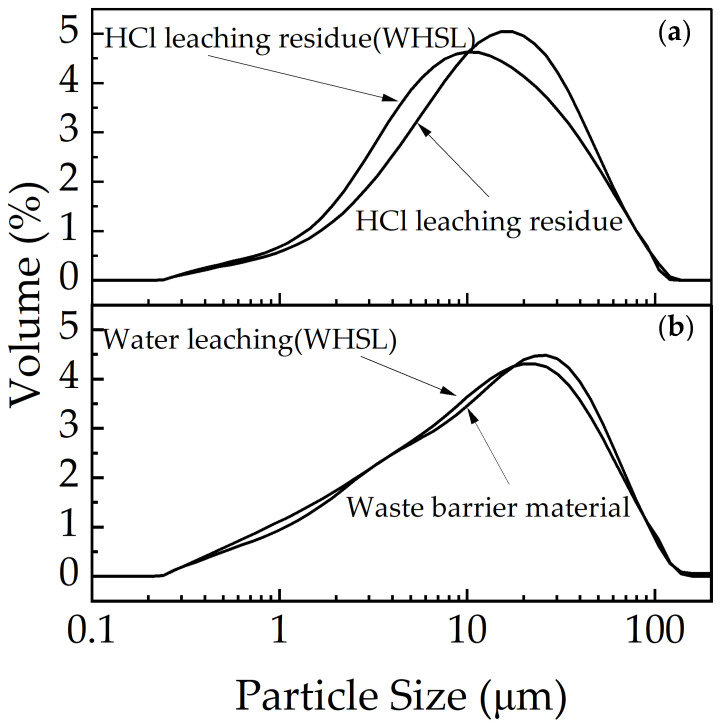
Particle size results: (**a**) HCl leaching (WHSL) and HCl leaching, (**b**) water leaching (WHSL), and waste barrier material.

The particle size distribution of the waste barrier material and its leaching residue is presented in Figure 10. Following water leaching (WHSL), the particle size of the waste barrier material slightly decreases and shifts towards the left (Figure 10b). In contrast, after HCl leaching, the particle size of the residue (Figure 10a) shows a more significant leftward shift, with the residue after HCl treatment (WHSL) exhibiting the most notable reduction.

X-ray diffraction (XRD) and Fourier-transform infrared (FTIR) spectroscopy confirm the formation of silica gel in the HCl-treated residue. The aggregation degree of precipitated hydrated silica particles significantly influences the structural properties of the leaching residue and thus the subsequent filtration performance. In this regard, the porosity in both the waste barrier material and residue is assessed through the measurement of oil absorption capacity, specifically using DBP (dibutyl phthalate) adsorption values, which reflect particle packing density and pore structure. Higher DBP absorption indicates looser particle packing and a more favorable pore structure [29].

**Figure 11:**
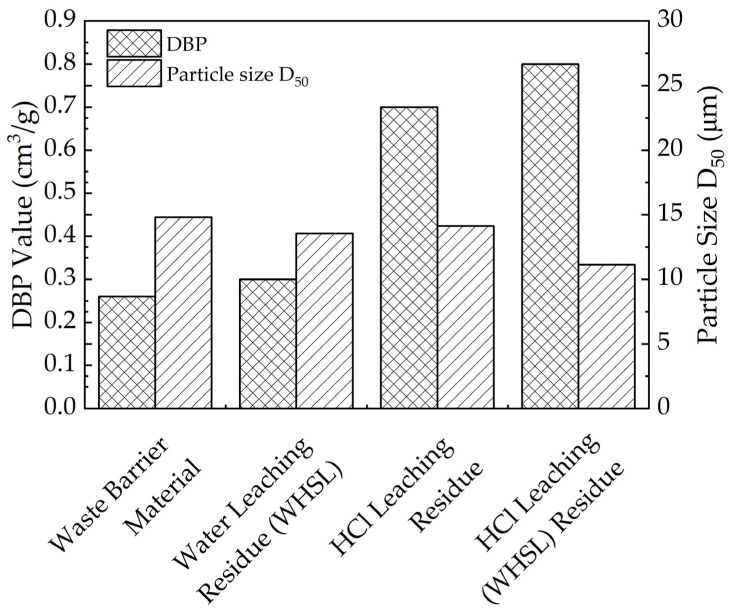
DBP value and particle size (D_50_) result from waste barrier material and leached residue in different conditions.

Figure 11 illustrates that the residue after water leaching (WHSL) shows a slight increase in DBP absorption (0.30 cm^3^/g) compared to the waste barrier material (0.26 cm^3^/g). This suggests that water leaching primarily dissolves NaF, with minimal effects on the silica structure. In contrast, the DBP values for residues after HCl leaching and HCl leaching (WHSL) are significantly higher (0.70 and 0.80 cm^3^/g, respectively), indicating that acid leaching promotes the formation of finer and more loosely aggregated silica gel particles [30,31]. Additionally, the D_50_ of the residue decreases, as anticipated, compared to the waste barrier material.

The zeta potential, which reflects the stability of a suspension, was measured to assess the potential for agglomeration [32]. The absolute value of zeta potential for the residues after HCl leaching and WHSL decreased to a range of 29–35 mV, compared to 43 mV for the waste barrier material, suggesting that smaller particle sizes (Table 2) in the residues are more prone to agglomeration [33].

#### 3.5.4. Proposed Mechanism

Water leaching dissolves approximately 50% of the fluoride ions, creating grooves and cracks on the material’s surface and enhancing porosity. Notably, the nepheline in the residue remains largely unaffected due to its stable structure. Conversely, during subsequent acid leaching, hydrochloric acid selectively extracts lithium from aluminum and silicon components, producing silica gel and aluminum hydroxide fluoride colloids through dissolution–precipitation reactions. These processes form a porous structure with finer, more loosely aggregated silica gel particles. Acid leaching also refines particle size (D50 decreases from 14.8 μm to 11.1 μm) and reduces surface potential (from 43 mV to 29–35 mV), facilitating colloidal aggregation. Therefore, the synergistic effect of water leaching reduces acid consumption and enhances the efficiency of acid leaching. Overall, WHSL achieves selective extraction of lithium from aluminum and silicon without HF emission.

## 4. Summary and Conclusions

The waste barrier material, one of the primary components of electrolytic cell overhaul slag, contains high concentrations of Li. While HCl leaching is traditionally employed for Li extraction, this method poses significant hazards. Therefore, a WHSL approach is proposed. In this process, water leaching (WHSL) extracts fluoride and water-soluble substances into the leachate, thereby preventing direct contact between high concentrations of fluoride and acids in the solution, as well as the neutralization of acids by alkaline liquids. Consequently, acid consumption and the generation of HF gas are significantly reduced. Furthermore, while a majority of the lithium is leached, the minimum amount of aluminum and silicon enters the leaching solution during the HCl leaching step (WHSL). This dual-step approach effectively minimizes both acid consumption and HF gas emissions, thereby conserving resources and mitigating environmental impact. A systematic investigation of the influencing factors during the leaching process identified optimal leaching conditions. Comprehensive analyses were conducted using ICP and fluoride ion meters for leachate elemental characterization, alongside XRD, SEM, FTIR, particle size analysis, and zeta potential measurements for residue characterization. Future research will focus on mechanistic investigations using XRD, SEM-EDS, and molecular dynamics simulations to evaluate the thermodynamic and kinetic processes behind silica gel aggregation. The main findings at this stage are outlined below.

1. WHSL achieved selective Li extraction without HF emission.
2. In water leaching, an increased temperature and liquid-to-solid ratio accelerate Li and F extraction. Similarly, in HCl leaching, a higher HCl concentration and liquid-to-solid ratio enhance Li, Al, and Si leaching.
3. SEM, particle size, and DBP analyses reveal increased surface roughness due to phase dissolution and reprecipitation, indicating enhanced porosity.
4. The waste barrier material primarily consists of nepheline (NaAlSiO_4_), corundum (Al_2_O_3_), sodium fluoride (NaF), and silicon (Si) phases. After leaching, aluminum hydroxide fluorite ((Al_2_(OH)_0.46_F_0.54_)6(H_2_O)) and amorphous silica predominated, while unreacted nepheline persisted.

## Figures and Tables

**Table 1:** Main chemical element composition of waste barrier material (wt.%).

Element	Al	Ca	Fe	K	Li	Na	Mg	F	Si
Content%	9.95	1.08	1.71	1.75	1.24	20.7	0.24	16.85	14.18

**Table 2:** Zeta potential result.

	pH	Zeta Potential, mv	Std, mv
Waste barrier material	8.96	−42.7	2.11
HCl leaching	6.51	−32.1	2.01
Water leaching (WHSL)	7.67	−35.1	2.36
HCl leaching (WHSL)	6.08	−29.7	2.25

## Data Availability

The original contributions presented in this study are included in the article. Further inquiries can be directed to the corresponding author.
